# M2 microglial small extracellular vesicles reduce glial scar formation *via* the miR-124/STAT3 pathway after ischemic stroke in mice

**DOI:** 10.7150/thno.48761

**Published:** 2021-01-01

**Authors:** Zongwei Li, Yaying Song, Tingting He, Ruoxue Wen, Yongfang Li, Tingting Chen, Shuxian Huang, Yongting Wang, Yaohui Tang, FanXia Shen, Heng-Li Tian, Guo-Yuan Yang, Zhijun Zhang

**Affiliations:** 1 Shanghai Jiao Tong Affiliated Sixth People's Hospital, School of Biomedical Engineering, Shanghai Jiao Tong University, Shanghai 200030, China.; 2 Department of Neurology, Renji Hospital of Shanghai Jiao Tong University, No. 160 Pujian Rd, Shanghai 200127, China.; 3 Shanghai Jiao Tong Affiliated Sixth People's Hospital, Shanghai Jiao Tong University, Shanghai 200030, China.; 4 Department of Neurology, Ruijin Hospital, School of Medicine, Shanghai Jiao Tong University, Shanghai 200025, China.

**Keywords:** astrocyte, small extracellular vesicles, glial scar, ischemic stroke, microglia

## Abstract

**Rationale:** Glial scars present a major obstacle for neuronal regeneration after stroke. Thus, approaches to promote their degradation and inhibit their formation are beneficial for stroke recovery. The interaction of microglia and astrocytes is known to be involved in glial scar formation after stroke; however, how microglia affect glial scar formation remains unclear.

**Methods:** Mice were treated daily with M2 microglial small extracellular vesicles through tail intravenous injections from day 1 to day 7 after middle cerebral artery occlusion. Glial scar, infarct volume, neurological score were detected after ischemia. microRNA and related protein were examined in peri-infarct areas of the brain following ischemia.

**Results:** M2 microglial small extracellular vesicles reduced glial scar formation and promoted recovery after stroke and were enriched in miR-124. Furthermore, M2 microglial small extracellular vesicle treatment decreased the expression of the astrocyte proliferation gene signal transducer and activator of transcription 3, one of the targets of miR-124, and glial fibrillary acidic protein and inhibited astrocyte proliferation both *in vitro* and *in vivo*. It also decreased Notch 1 expression and increased Sox2 expression in astrocytes, which suggested that astrocytes had transformed into neuronal progenitor cells. Finally, miR-124 knockdown in M2 microglial small extracellular vesicles blocked their effects on glial scars and stroke recovery.

**Conclusions:** Our results showed, for the first time, that microglia regulate glial scar formation *via* small extracellular vesicles, indicating that M2 microglial small extracellular vesicles could represent a new therapeutic approach for stroke.

## Introduction

Glial scarring is a major obstacle for axonal regeneration and functional recovery at the late stage of ischemic stroke 1. As it is an important therapeutic target for axonal regrowth and functional recovery after stroke 2, a good understanding of the regulation of glial scar formation is fundamental to developing safe interventions for stroke. Glial scarring is characterized by the proliferation of microglia and reactive astrocytes 3, which exhibit enlarged soma, thickened astrocytic processes, and increased glial fibrillary acidic protein (GFAP) immunoreactivity. The activation of signal transducer and activator of transcription 3 (STAT3), a canonical regulator of astrogliosis 4, in astrocytes promotes GFAP expression and leads to severe astrogliosis after brain injury 5. Conversely, STAT3 inhibition can reduce brain damage and improve neurological outcome in different stroke models 6-8. Nevertheless, the cellular pathways mediated by STAT3 in glial scar formation after cerebral ischemia remain unclear.

Furthermore, microglia and astrocytes are known to cooperate reciprocally during glial scar formation, but few studies have shown how microglia regulate this process. Microglia, the resident innate immune cells of the brain, can dynamically survey the brain environment and play crucial roles in microenvironment maintenance, injury response, and pathogen defense 9, 10. After ischemic injury, microglia are the first respond to the damage and rapidly release inflammatory mediators that are either cytotoxic or cytoprotective. The different functions of the secreted mediators depend on the two different microglial phenotypes: anti-inflammatory (involving Type 2 [M2] microglia) and pro-inflammatory (involving Type 1 [M1] microglia). M2 microglia are regarded as protective cells that can contribute to tissue recovery after damage and secrete anti-inflammatory mediators such as interleukin-10 (IL-10), interleukin-4 (IL-4), interleukin-13 (IL-13), transforming growth factor-β, insulin growth factor-1, and various other neurotrophic factors 11. The interactions between microglia and astrocytes provide a regulatory system for glial scar formation after injury 12, 13; however, how microglia regulate glial scar formation and the role of M2 microglia in this process are unclear.

Small extracellular vesicles (sEVs) are key players in intercellular signaling 14 15, and sEV-enriched microRNAs (miRNAs) can be transferred to adjacent or distant recipient cells to participate in intercellular genetic regulatory activities 16. miRNAs can regulate the expression of a wide variety of genes at the post-transcription level by causing mRNA degradation or translational repression. Moreover, miRNAs derived from M2 microglial sEVs play an important role in pathological processes after brain injury 17, 18. Based on a previous analysis of miRNA microarray results, we showed that miR-124 levels increased significantly in M2 microglial sEVs, and was upregulated in the ischemic penumbra region at 72 h after transient middle cerebral artery occlusion (tMCAO) 19. Moreover, STAT3 is one of the direct targets of miR-124 20, and phosphorylated STAT3 (p-STAT3) is an important regulator of astrocyte activation even after post-ischemic astrogliosis 5. As microglia and astrocytes tightly cooperate and reciprocally modulate the formation of glial scars, here, we tested the hypothesis that whether microglia affect astrogliosis *via* miR-124 of sEV.

In this study, BV2 cells are substituted for microglia to obtain enough sEV, we found that sEVs derived from M2 BV2 cells reduced glial scar formation and improved neurological functional recovery after stroke *via* the miR-124/STAT3 signal pathway.

## Materials and methods

### Experimental design

All animal procedures were carried out in accordance with the guidelines of the Institutional Animal Care and Use Committee of Shanghai Jiao Tong University, Shanghai, China and reported according to the ARRIVE guidelines. Eighty adult (aged approximately 16 weeks) male ICR mice (Jiesijie, Shanghai, China) weighing 25-28 g were used and randomly divided into the sham, phosphate-buffered saline (PBS), M2-sEV, and miR-124 knockdown (miR-124-kd) groups (12 mice in the PBS group and 15 each in the sham, M2-sEV, and miR-124-kd groups) by an investigator blinded to the experimental design. M2 BV2 cell miR-124 was knocked down using the miR-124-kd lentivirus (OBiO, Shanghai, China; virus titer, 109 TU/mL) or lentiviral vector alone, and the exosomes isolated from vector alone cells were considered as the control of miR-124-kd. Mice were housed with free access to food and water under a 12 h light-dark cycle (light on at 8:00 and off at 20:00). The PBS, M2-sEV, and miR-124-kd mice underwent 90 min of tMCAO. Animals in the M2-sEV and miR-124-kd groups were treated daily with sEVs from M2 BV2 cells through tail intravenous injections (4 µg/kg) 21 from day 1 to day 7 after tMCAO. Mice in the PBS group were treated with equal volumes of PBS in the same manner. Aldh1l1- CreERT2:Ai14 (gift from Korea Advanced Institute of Science and Technology) transgenic mice were used to trace reactive astrocyte transdifferentiation. Bromodeoxyuridine (BrdU) (Sigma-Aldrich, B2531, San Louis, CA) was intraperitoneally injected into all mice (50 mg/kg per day) beginning on day 7 after ischemia 19.

### tMCAO

tMCAO surgery was performed as previously described 22 . Briefly, mice were anesthetized using 1.5% isoflurane in 30% O_2_/68.5% NO under spontaneous breathing conditions. An incision was made at the midline of the neck, and the middle cerebral artery was blocked by gently inserting a 6-0 silica-coated nylon suture (Covidien, Saint Louis, MA) from the left external carotid artery to the original middle cerebral artery. Successful occlusion was confirmed using laser Doppler flowmetry (moorVMS-LDF1-HP, Moor Instruments) as a decline in regional blood flow of more than 80% compared to that at baseline. After 90 min of transient ischemia, the suture was withdrawn, and reperfusion was allowed. Animals with successful occlusion and reperfusion were included in the experiments and sacrificed at 7 or 14 days after tMCAO for western blotting, gene expression, and immunostaining analysis and at day 14 after tMCAO for atrophy volume analysis.

### Astrocyte culture

Primary astrocyte cultures were prepared from postnatal ICR mice (Jiesijie, Shanghai, China). Briefly, the cortex was isolated from the brain under a microscope and then trypsinized at 37 °C for 12 min. After centrifugation, cell pellets were suspended in culture medium (90% Dulbecco's Modified Eagle medium [DMEM], 10% fetal bovine serum [FBS], 100 U/mL penicillin G, and 100 mg/mL streptomycin sulfate) and filtered through 70 μm strainers (Millipore, Billerica, MA), then plated onto poly-D-lysine coated dishes. Cell cultures were maintained in an incubator at 37 °C at 95% humidity in 5% CO_2_. One day later, the medium was replaced with fresh culture medium; subsequently, the medium was replaced every 3 days.

### Brain atrophy volume measurement

Mouse brains were perfused with 0.1 mol/L PBS and then with 4% paraformaldehyde (PFA). Brain samples post-fixed overnight in 4% PFA were dehydrated in 30% sucrose until they sank to the bottom of the liquid and then stored at -80 °C. Subsequently, the samples were cut into 30 μm sections from the anterior commissure to the hippocampus and stained with 1% cresyl violet. The atrophy area ΔS was calculated as the contralateral area minus the ipsilateral area, and the atrophy volume was calculated as: V = 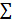 h / 3 [ ΔSn + 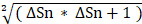 + ΔSn+1], where “h” represents the thickness between the two sections 23.

### Neurological function tests

Neurobehavioral tests were conducted at 7 and 14 days after tMCAO by an investigator blinded to the experimental design using the modified neurological severity score (mNSS) as previously described 24. The mNSS, which ranges from 0 to 14, is obtained based on a comprehensive assessment of neurological functions, including motor skills, balance, and reflexes. A higher behavioral outcome score represents more severe ischemic injury.

#### Rotarod test

An operator blinded to group assignment conducted the rotarod test on day 0 before tMCAO as the baseline and then on days 7 and 14 after tMCAO. The duration of time for which each mouse stayed on the rod was recorded 25.

#### Corner test

The corner test assesses sensory and motor dysfunction after injury to the cortex and striatum in mice. In this study, we used two hardwood boards (30×20×1 cm each) connected to each other at an angle of 30° from the edge. Mice were placed facing the 30° angle corner for testing. As the mice walked into the corner, facial contact with the board made them turn towards the open ends of the two boards, and this turning behavior to either side was recorded. Twenty turns were evaluated in one trial, and the percentage of right turns was calculated. Tests were conducted before performing ischemic surgery to establish a baseline. Subsequently, mice were tested 7 and 14 days after tMCAO 26.

#### Immunohistochemistry

The sample collection procedure was the same as that for brain atrophy volume measurement. Thereafter, cells or brain sections were fixed in 4% PFA for 10 min, permeabilized in 0.3% Triton X-100 for 10 min, and then incubated in 10% bovine serum albumin (BSA) for 1 h at room temperature to block non-specific binding. Finally, they were incubated overnight at 4 °C with primary antibodies against the following as appropriate: GFAP (1:500; MAB3402, Millipore, Billerica, MA), BrdU (1:200; sc-56255, Santa Cruz, CA), Iba-1 (1:200; NB100-1028, Novusbio, Lieeleton), arginase (1:500; sc-271430, Santa Cruz, CA), CD206 (1:500, R&D System, AF2535), nestin (1:500, ab81462, Abcam, Cambridge, MA), and Sox2 (1:500, ab97959, Abcam, Cambridge, MA). For BrdU staining, brain sections were incubated with 2 M hydrochloric acid at 37 °C for 30 min and neutralized with 0.1 M sodium borate (pH 8.5) for 10 min after permeabilization and before blocking. After removing excess primary antibodies, the samples were incubated with fluorescence-tagged secondary antibodies at room temperature for 1 h. Excess secondary antibodies were removed, and the samples were incubated with 4, 6-diamidino-2-phenylindole (DAPI, BIOTIUM-23002, Life Technologies) for 10 min at room temperature. The samples were then imaged using laser scanning confocal microscopy (Leica, Soloms, Germany) 27.

#### Astrocyte analysis

Four brain sections at every 200 µm interval were selected per animal for immunostaining, and five peri-infarct areas of each brain section were randomly chosen for confocal imaging. The longest process length and number of processes per astrocyte around the scar were measured. Approximately 60 astrocytes per mouse were counted. Image J software (NIH, Bethesda, MD, USA, RRID: SCR_003070) was used for image analysis. Imaging and image analysis were performed by a blinded investigator 2.

### Western blot analysis

Fresh mouse brains were collected and sectioned into 2 mm slices that included the ischemic core and peri-infarct areas in a mouse brain mold (RWD Company, China), and peri-infarct areas were collected from the ipsilateral cerebral hemisphere. For cell and brain tissue samples, lysates were prepared using RIPA lysis buffer (Millipore, Billerica, MA) supplemented with a protease inhibitor cocktail 28. Protein concentrations were determined using the Pierce^TM^ BCA Protein Assay Kit (ThermoFisher Scientific, NY). Equal amounts of protein (30 µg) were separated by standard sodium dodecyl sulfate polyacrylamide gel electrophoresis and then electrotransferred onto polyvinylidene fluoride membranes, which were blocked with 5% non-fat milk at room temperature for 1 h and incubated overnight at 4 °C with primary antibodies against the following as appropriate: GFAP (1:1000; MAB3402, Millipore, Billerica, MA), TSG101 (1:500; ab30871, Abcam, Cambridge, MA), CD63 (1:1000; sc-15363, Santa Cruz, MA), nestin (1:1000; MAB353, Millipore, Billerica, MA), Sox2 (1:1000; ab97959, Abcam, Cambridge, MA); p-STAT3 (1:1000; 9145S, Cell Signaling Technology, Beverly, MA), STAT3 (1:1000; 12640S, Cell Signaling Technology, Beverly, MA), Notch1 (1:1000; ab8925, Abcam, Cambridge, MA), and β-actin (1:1000; MA5-15739, Invitrogen, Carlsbad, CA). After removing excess primary antibodies, the membranes were incubated with appropriate horseradish peroxidase-conjugated secondary antibodies for 1 h at room temperature. Subsequently, the membranes were incubated with ECL solution (ThermoFisher Scientific, NY, USA), and the signal was detected using an imaging system (Tanon 5200, Shanghai Tianneng Technology Co., Ltd, China).

### Total RNA extraction and quantitative real-time PCR

Brain tissue samples were extracted from the glial scar region under a microscope (Leica, Solms, Germany). Total RNA was extracted from brain or cell samples using the TRIzol reagent (Invitrogen, Carlsbad, CA, USA). RNA (1 µg) was reverse transcribed to cDNA using a Hifair® II First-strand cDNA Synthesis Kit (Yeasen Biotech, Shanghai, China). mRNA expression levels were quantified using a SYBR Green Master Mix (Exiqon, Vedbaek, Denmark), and Ct values for each sample and gene were normalized with respect to glyceraldehyde 3-phosphate dehydrogenase. The expression of miRNA was tested using a fast real-time PCR system (7900 HT, ABI, Foster City, CA) and the appropriate miRNA oligonucleotide primers (Qiagen, Hilden, Germany). The fold-change values were calculated by normalizing with respect to the control samples. PCR amplification was performed for 40 cycles, and the data were collected using SDS software (Applied Biosystems, Foster City, CA). The sequences of the mRNA oligonucleotide primers were used are listed in the table follows:

### Oxygen-glucose deprivation (OGD) and re-oxygenation model

Primary astrocytes were plated into six-well plates at a density of 5×10^5^ cells per well. After pretreatment with or without M2-sEVs (10 μg/mL) for 24 h, astrocytes, except those in the control group, were rinsed once with PBS and then maintained in DMEM without glucose in an anaerobic chamber infused with a gas mixture containing 0% O_2_, 5% CO_2_, and 95% N_2_ at 37 °C. After incubation for 3 h, the culture medium was replaced, and cells were re-oxygenated for 24 h to mimic *in vivo* reperfusion-like conditions.

### sEV isolation and labeling

sEV-depleted FBS obtained after ultracentrifugation at 100,000 × g for 16 h at 4 °C was used for murine M2 BV2 cell culture. sEVs were purified from the cell culture supernatant of M2 BV2 cells as follows. M2 BV2 cCells were induced using IL-4 (20 ng/mL) and cultured in 95% DMEM with 5% FBS, 100 U/mL penicillin G, and 100 mg/mL streptomycin sulfate. When the cells reached 90% confluence, supernatant was harvested and centrifuged at 300× g at 4 °C for 10 min to remove any residual cells. The supernatants were then centrifuged at 2000 × g at 4 °C for 10 min and 10,000 × g at 4 °C for 30 min to remove cell debris. Subsequently, exosomes were collected through ultracentrifugation at 100,000 × g at 4 °C for 70 min. The pellets were suspended in PBS, ultracentrifugation was repeated, and the purified exosomes were resuspended in PBS and diluted to 1 µg/mL for further experiments.

Fluorescence labeling of sEVs was performed using the PKH26 Kit (Sigma-Aldrich, San Louis, CA) according to the manufacturer's protocol. sEV suspensions (100 µL) were added to 1 mL diluent C and incubated with sEV solution for 4 min. To remove excess dye, 0.5% BSA/PBS was added to the sample. Then, the labeled sEVs were washed at 100,000 × g at 4 °C for 1 h, and the sEV pellets were diluted in 100 μL PBS for further experiments.

### Electron microscopy and nanoparticle tracking analysis (NTA)

Transmission electron microscopy (TEM; Phillips CM120) was used to detect M2 BV2 cell-derived sEVs. sEVs were fixed in 2% paraformaldehyde on formvar-carbon-coated grids, and the membranes were covered for 30 min in a dry environment. Thereafter, the samples were washed with 100 μL of PBS, and grids were transferred to 50 μL 1% glutaraldehyde for 10 min before being transferred to 100 µL distilled water for 10 min. These steps were repeated seven times, after which the grids were dried and observed by TEM at 200 kV.

NTA was used to track the sEV diameter and number of particles. To provide representative size distribution profiles, data obtained from NTA were averaged within each sample across the video replicates and then averaged across samples. These distribution profiles were normalized to the total nanoparticle concentrations.

### Cell viability assay

Cell growth was determined using the Cell Counting Kit-8 Solution (Dojindo, Kumamoto, Japan). Briefly, astrocytes were plated into 96-well plates at a density of 1×10^4^ cells per well. When the cells reached 60% confluence, they were pretreated with or without M2-sEVs (10 μg/mL) for 24 h. The cells were then subjected to OGD for 3 h and re-oxygenation for another 24 h. Thereafter, the culture medium was replaced with DMEM containing 10% CCK-8 solution, and cells were incubated for 2 h at 37 °C in 5% CO_2_. The absorbance at 450 nm was measured using a microplate reader (BioTek, Winooski, VT).

### Scratch migration assay

Astrocytes were plated into six-well plates at a density of 5×10^5^ cells per well. When cells reached 90% confluence, they were pretreated with or without M2-sEVs (10 μg/mL). After 24 h of treatment, a scratch was made through the confluent monolayer using a sterile 200 μL plastic micropipette tip. Cells were washed twice with PBS and then subjected to OGD followed by re-oxygenation. The scratch gap area was viewed 24 h later under a microscope (Leica, Solms, Germany).

### Statistical analysis

Results are presented as mean ± standard error of mean (SEM), and statistical comparisons were assessed using Student's t-test or one-way analysis of variance followed by the post hoc least-significant difference test. *p* <0.05 was considered significant, and GraphPad Prism 5 (GraphPad Software Inc.) was used for all statistical analysis.

## Results

### Characterization of M2 BV2 cells induced by IL-4 and identification of sEVs derived from M2 BV2 cells

**Figure 1:**
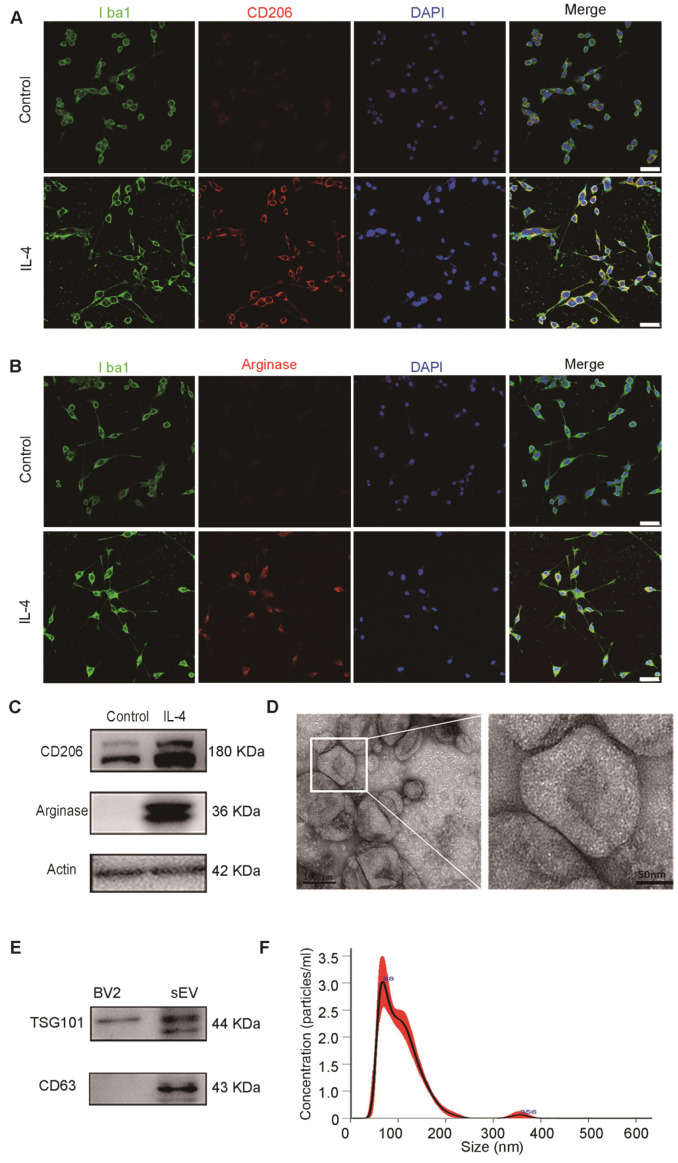
** Characterization of M2 BV2 cells induced by IL-4 and identification of sEVs derived from M2 BV2 cells.** (**A, B**) Representative images of BV2 cells immunostained for Iba-1 (green), CD206 (red), and arginase (red). Cultured systems were treated with 0 or 20 ng/µL IL-4. Cell nuclei were counterstained with DAPI. Scale bar = 50 µm. (**C**) Western blotting analysis of CD206 and arginase expression in BV2 cells after 0 or 20 ng/µL IL-4 treatment. (**D**) Representative electron microscopy images showing the phenotype of M2-sEVs. Left image scale bar = 100 nm, right image scale bar = 50 nm. (**F**) NTA of M2-sEVs isolated by ultracentrifugation from M2 BV2 cells. Data represent the average size distribution profile of three samples and each purification normalized to the total nanoparticle concentrations. Data for each sample was derived from three different videos and analyses. (**G**) Western blotting analysis of TSG101 and CD63 levels in M2 BV2 cells and M2-sEVs.

To identify the characteristics of M2 BV2 cells induced by IL-4, we used immunostaining for Iba1, a microglial marker, CD206 and arginase, M2-polarized microglial marker. We found that BV2 cells expressed microglia-specific maker Iba1, CD206 and arginase were expressed in IL-4 induced BV2 cells, whereas expression was almost undetectable in control cells **(Figure 1A-B)**. At the same time, we also used western blotting to detect CD206 and arginase expression in BV2 cells with or without IL-4 treatment. The results showed that CD206 and arginase were expressed at higher levels in IL-4 treated BV2 cells compared to those in the controls, suggesting that IL-4 successfully induced M2-polarized cells **(Figure 1C)**.

To examine the physical characteristics of M2-sEVs derived from M2 BV2 cells, we used TEM and NTA and found that the sEVs had a typical cup-shaped membrane vesicle morphology with a diameter of 30-120 nm **(Figure 1D, 1F)**. Furthermore, we examined the sEV-specific markers TSG101 and CD63 in cells and sEV lysates using western blot analysis. TSG101 and CD63 were highly expressed in sEV fractions, while TSG101 levels were very low in cell lysates **(Figure 1E)**.

### M2-sEVs attenuated glial scar formation and astrocyte activation after tMCAO

**Figure 2:**
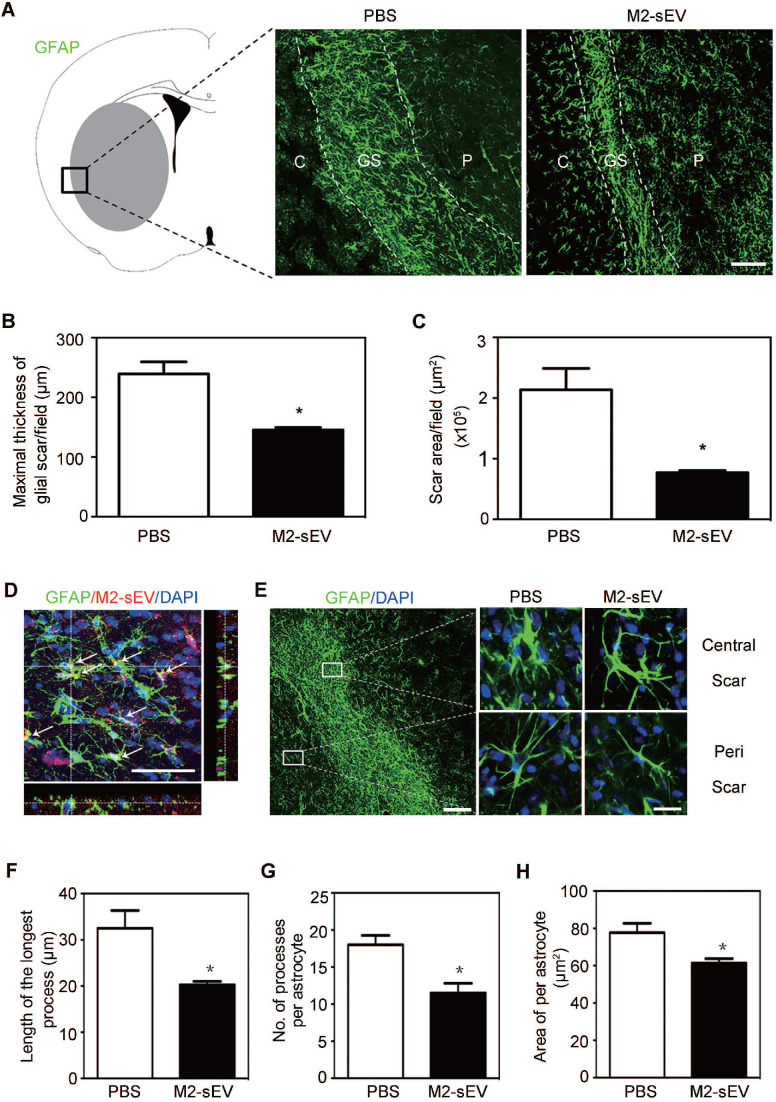
** M2-sEVs inhibited glial scar formation and attenuated astrocyte activation in the mouse brain after tMCAO.** (**A**) Representative images of GFAP (green) immunostaining in the PBS and M2-sEV groups 14 days after tMCAO. C: infarct center, GS: glial scar, P: peri-infarct area. Scale bar = 100 µm. (**B, C**) Statistical analysis of the glial scar areas and maximal scar thickness per field in the PBS and M2-sEV groups at 14 days after tMCAO (n = 10). (**D**) Representative images of GFAP (green) and PKH26 (red) immunostaining in the tMCAO mouse brain. The yellow color indicates sEVs in astrocytes. The cell nuclei were counterstained with DAPI. Scale bar = 50 µm. (**E**) Left panel: Locations of central and peri-scarring observed using GFAP (green) immunostaining in mouse brains 14 days after tMCAO. Scale bar = 100 µm. Right panel: Representative images of magnified photographs of astrocyte morphology in the central and peri-scar areas of the PBS and M2-sEV groups. Scale bar = 10 µm. (**F-I**) Statistical analysis of the length of the longest process, the number of processes, and swallow area per astrocyte in the control and M2-sEV mouse brains 14 days after tMCAO (n = 10). The data are presented as mean ± SEM. *p < 0.05.

Alterations in intermediate filament (IF) protein expression is a recognized feature of reactive astrogliosis. 1. In order to examine the effects of M2-sEVs on glial scar formation, we performed immunofluorescent staining of GFAP, a mature astrocyte IF protein, in ischemic mouse brains with or without M2-sEV treatment. The results showed that scar area and maximal scar thickness decreased in the M2-sEV group compared to that in the control group, suggesting that M2-sEVs could attenuate glial scar formation after ischemic stroke **(Figure 2A-C)**.

To determine whether injected M2-sEVs were taken up by astrocytes, we used PKH26, a red fluorescent dye for sEVs, to label M2-sEVs, which were then injected into ischemic mice. Thereafter, GFAP immunostaining was performed to observe astrocytes. We found that M2-sEVs accumulated around the nucleus of astrocytes **(Figure 2D)**, suggesting that astrocytes were able to phagocytose M2-sEVs actively. In addition, M2-sEVs could alter astrocyte activation in the ischemic mouse brain. The swollen volume of astrocytes was smaller in the glial scar center of the M2-sEV group than in the control group **(Figure 2E)**. Furthermore, we checked the longest process length, process number, and swallow area of each astrocyte around the scar. The results showed that the longest process length, number of processes, and swallow area per astrocyte decreased in the M2-sEV group compared to those in the control group** (Figure 2F-H)**. Taken together, these results indicated that M2-sEVs could attenuate glial scar formation by reducing astrocyte activation after ischemia.

### M2-sEVs attenuated astrocyte proliferation and migration after tMCAO

**Figure 3:**
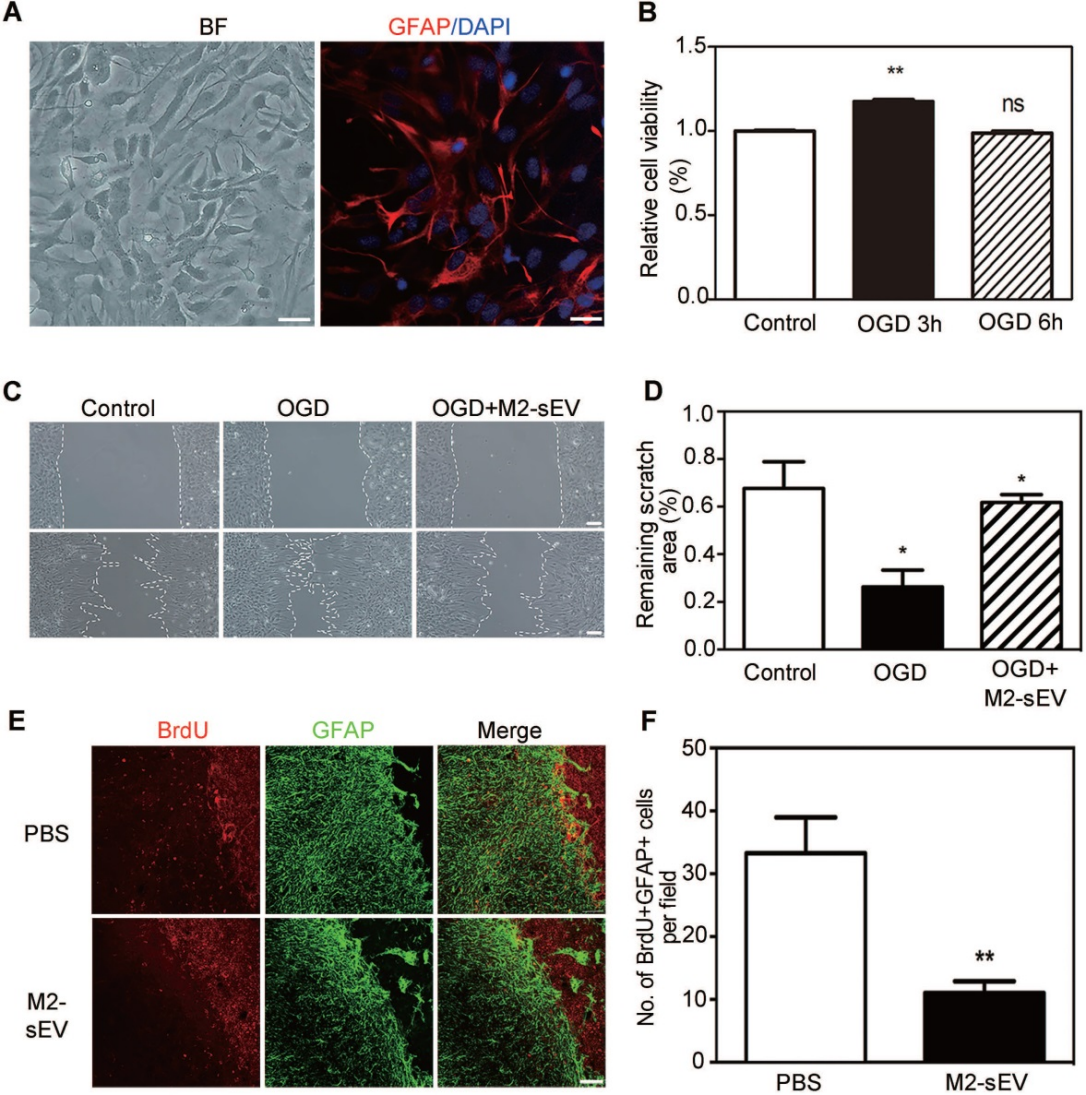
** M2-sEVs inhibited the proliferation and migration of astrocytes after tMCAO.** (**A**) Representative image of primary astrocytes in bright field and Immunostaining of GFAP (red) with DAPI counterstaining for cell nuclei. Left image scale bar = 50 µm, right image scale bar = 10 µm. (**B**) CCK-8 assay of cultured astrocytes after 0, 3, and 6 h of OGD treatment. The absorbance at 450 nm was normalized to and expressed as a percentage of the control values. (**C**) Scratch assay of cultured astrocytes after 0 or 3 h of OGD treatment and reoxygenation for 24 h. 10 µg/mL M2-sEVs were pretreated for 24 h before OGD treatment. Scale bar = 50 µm. (**D**) Statistical analysis of the remaining scratch area rates in the control, OGD, and OGD+M2-sEV groups at 24 h. OGD treatment promoted astrocyte migration to the scratch, while M2-sEVs inhibited the astrocyte migration. (**E**) Representative images of GFAP (green) and BrdU (red) immunostaining in the PBS and M2-sEV groups 14 days after tMCAO. Scale bar = 50 µm. (**F**) Statistical analysis of the number of GFAP^+^/BrdU^+^ cells in the PBS and M2-sEV groups 14 days after tMCAO (n = 12). Data are presented as mean ± SEM. *p < 0.05, **p < 0.01.

The migration and proliferation of reactive astrocytes have been shown to be involved in glial scar formation in a coordinated manner 29. To examine the effect of M2-sEVs on the migration of astrocytes, we established an *in vitro* cellular model to mimic ischemia-reperfusion injury. Primary astrocytes were identified using bright field microscopy and immunofluorescent staining of GFAP. We found that more than 95% of the cultured primary cells were GFAP positive** (Figure 3A)**. In order to assess the survivability of astrocytes under OGD conditions, we examined astrocyte viability using the CCK-8 assay after different durations of OGD treatment with 24 h reoxygenation. The results showed that cell viability significantly increased after 3 h of OGD treatment compared to the control condition **(Figure 3B)**. Since the CCK-8 assay reflects the activity of mitochondrial dehydrogenase, our results suggested that astrocytes had a stronger proliferation capacity after 3 h of OGD treatment. Therefore, we chose 3 h time point for subsequent OGD experiments.

To determine whether the decrease in glial scar formation was due to the attenuation of astrocyte migration induced by M2-sEVs, we performed a cell scratch assay. The 10 μg/mL M2-sEV dosage was determined on the basis of a gradient experiment (data not shown). The results showed that the remaining scratch area was larger in the M2-sEV group than in the control group **(Figure 3C-D),** suggesting that M2-sEVs reduced the migration of astrocytes. Next, we examined astrocyte proliferation by using GFAP and BrdU immunostaining of the ischemic mouse brain. The results showed that the number of GFAP and BrdU double-positive cells decreased in the M2-sEV group compared to that in the control group** (Figure 3E-F)**, suggesting that M2-sEVs also reduced astrocyte proliferation. These results indicated that the decrease in glial scar formation induced by M2-sEVs was due to the attenuation of astrocyte migration and proliferation.

### M2-sEVs promoted astrocyte to neural progenitor transition after tMCAO

**Figure 4:**
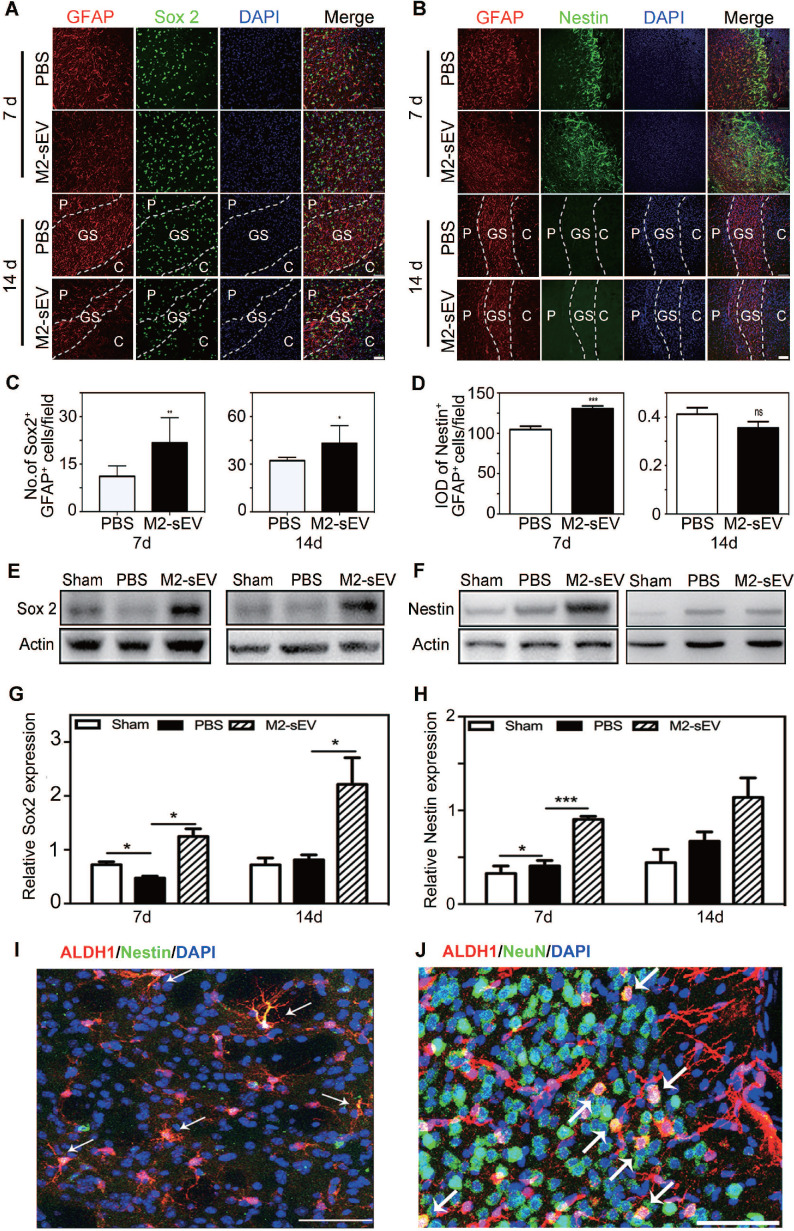
** M2-sEVs promoted the transition of astrocytes to neural progenitors in the mouse brain after tMCAO.** (**A**) Representative images of GFAP (red) and Sox 2 (green) immunostaining in the PBS and M2-sEV groups at 7 days and 14 days after tMCAO. Scale bar = 50 µm. (**B**) Representative images of GFAP (red) and nestin (green) immunostaining in the control and M2-sEV groups. Scale bar = 50 µm. (**C**) Statistical analysis of the number of GFAP^+^/Sox 2^+^ cells in the PBS and M2-sEV groups after tMCAO (n = 12). (**D**) Statistical analysis of the integrated optical density of GFAP^+^/nestin^+^ cells in the control and M2-sEV groups after tMCAO (n = 12). (**E, F**) Western blotting analysis of Sox 2 and nestin expression in the sham, PBS, and M2-sEV groups at 7 days and 14 days after tMCAO (n = 12). (**G, H**) Quantification of Sox 2 and nestin expression. (**I**) Representative images of Aldh1l1 (red) and nestin (green) in M2-sEV group at 7 days after tMCAO. Scale bar = 50 µm. (**J**) Representative images of Aldh1l1 (red) and NeuN (green) in M2-sEV groups at 14 days after tMCAO. Scale bar = 50 µm. Data are presented as mean ± SEM. **p < 0.01, ***p < 0.005.

The expression of Sox2 is sufficient to reprogram astrocytes to neuroblasts during central nervous system (CNS) injury 30. Sox2 is a transcription factor that is essential for maintaining pluripotency, while nestin is a typical marker of neural progenitors. We found higher reactive astrocyte expression of Sox2 and nestin in the peri-infarct area of the M2-sEV group than in the PBS group at 7 days after tMCAO. Sox2 was intensely expressed in the glial scar in the M2-sEV group, and nestin was almost undetectable in both the PBS and M2-sEV groups at 14 days after tMCAO **(Figure 4A-D)**. To further detect the expression levels of Sox2 and nestin in the ischemic brain, we performed a western blot assay. The results showed that Sox2 expression increased in the M2-sEV group after tMCAO. Nestin expression in the M2-sEV group increased during the first 7 days after ischemia and then decreased** (Figure 4E-H)**. To trace reactive astrocyte transdifferentiation, we used Aldh1l1- CreERT2:Ai14 transgenic mice. The results showed that nestin was co-expressed in Aldh1l1 tdTomato^+^ cells at 7 days after tMCAO, some Aldh1l1 tdTomato^+^ cells co-expressed NeuN at 14 days after tMCAO in M2-sEV group **(Figure 4I-J)**. Taken together, our results suggest that M2-sEV might induce a transition from reactive astrocytes to neural progenitors.

### M2-sEVs increased the expression of miR-124 in the ischemic mouse brain

**Figure 5:**
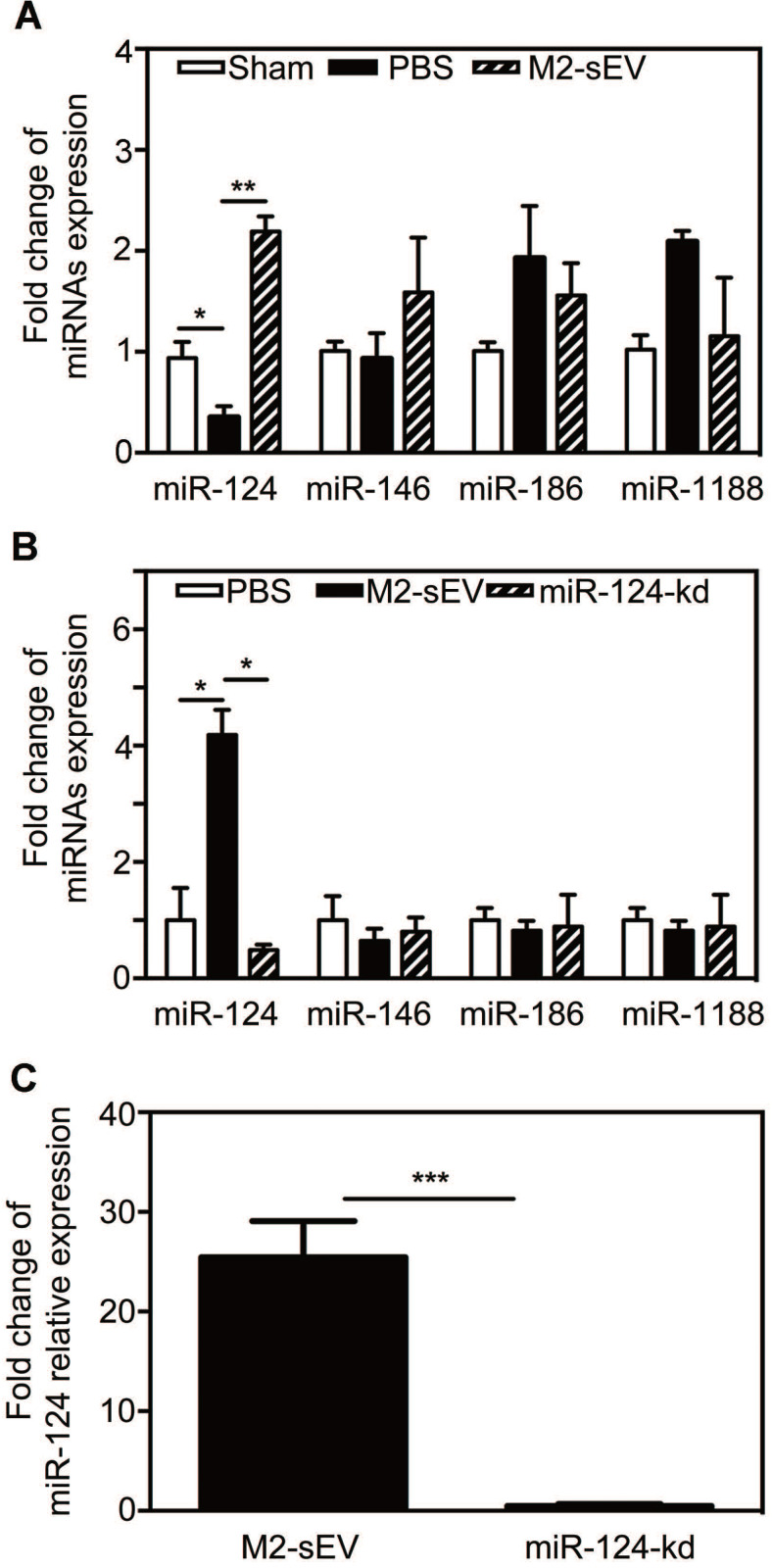
** M2-sEV improved miR-124 expression in the mouse brain after tMCAO.** (**A**) miR-124, miR-146, miR-186, and miR-1188 expression in the sham-, PBS -, and M2-sEV groups 7 days after tMCAO (n = 9). Data are presented as mean ± SEM. *p < 0.05, **p < 0.01. (**B**) miR-124, miR-146, miR-186, and miR-1188 expression in the PBS -, M2-sEV, - and miR-124-kd groups at 7 days after tMCAO (n = 9). (**C**) miR-124 expression in M2-sEV and miR-124-kd groups at 7 days after tMCAO. Data are presented as mean ± SEM. *p < 0.05, **p < 0.01, ***p < 0.005.

It has been well documented that miRNAs are enriched in sEVs and can be released to regulate the function of recipient cells. To determine whether the decrease in glial scar formation was due to M2-sEV miRNA, we chose miRNAs expressed in the brain and associated with the nervous system based on the results of a previous miRNA microarray analysis - miR-124, miR-146, miR-186, and miR-1188 - and assessed their expression in the brain at 7 days after tMCAO. The results showed that only miR-124 expression decreased after ischemia, and that M2-sEVs could significantly increase miR-124 expression **(Figure 5A)**. The expression of miR-146, miR-186, and miR-1188 did not differ among groups.

To explore the effects of M2-sEV miR-124 on glial scar formation, we used a miR-124-shRNA lentiviral vector to knock down miR-124 in M2 BV2 cells as previously described 19. We then used these sEVs to treat tMCAO mice and examined the miR-124, miR-146, miR-186, and miR-1188 expression to find that only miR-124 expression was downregulated in the ischemic mouse brain **(Figure 5B-C)**.

### Downregulation of miR-124 blocked the inhibitory effect of M2-sEVs on glial scar formation after tMCAO

**Figure 6:**
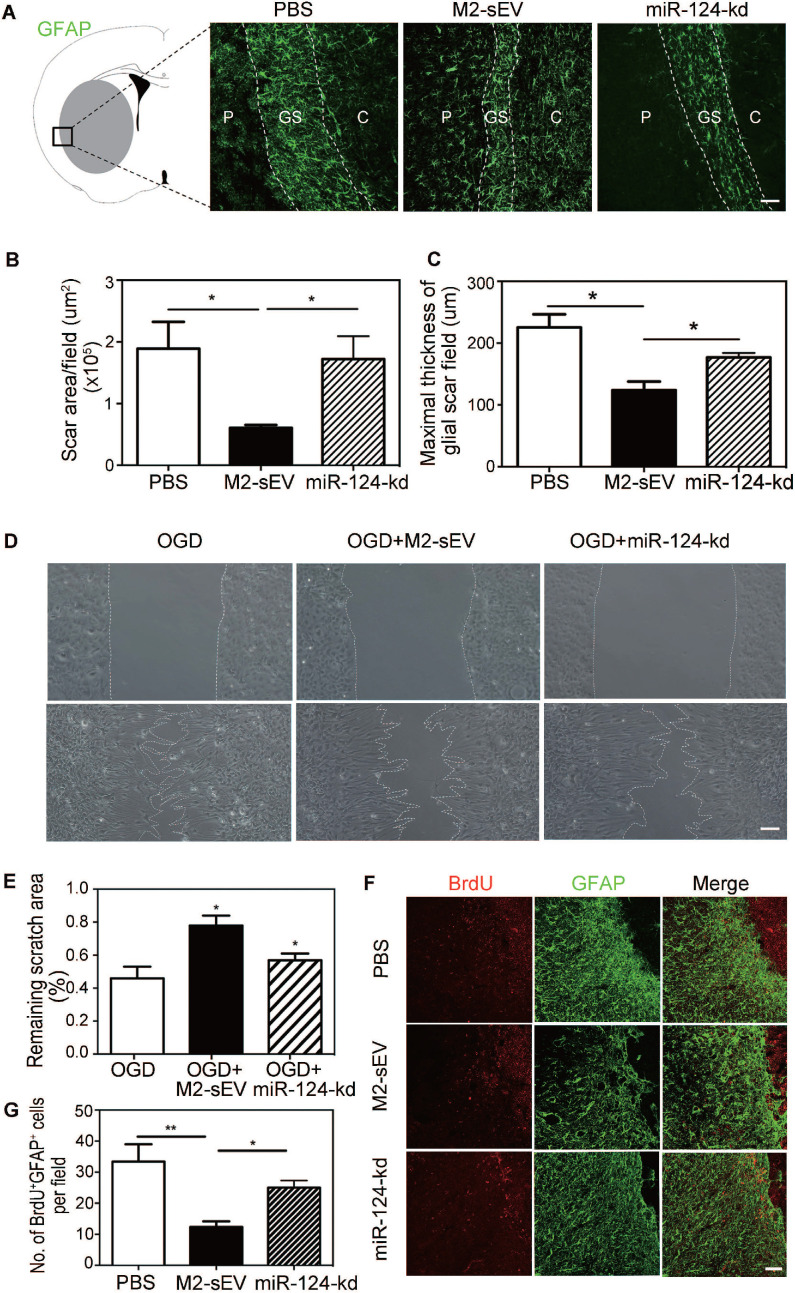
** Downregulation of miR-124 blocked the inhibitory effect of M2-sEVs on glial scar formation after tMCAO.** Representative images of GFAP (green) immunostaining in the PBS -, M2-sEV -, and miR-124-kd groups 14 days after tMCAO. C: infarct center, GS: glial scar, P: peri-infarct area. Scale bar = 100 µm. (**B, C**) Semi-quantification of the glial scar areas and maximal scar thickness per field in the PBS, M2-sEV, and miR-124-kd groups (n = 15). (**D**) Scratch assay results of cultured astrocytes after 3 h of OGD treatment and reoxygenation for 24 h. 10 µg/mL M2-sEVs as well as miR-124-kd M2-sEVs were pretreated for 24 h before OGD treatment. Scale bar = 50 µm. (**E**) The remaining scratch areas in the OGD, OGD+M2-sEV, and OGD+miR-124-kd groups at 24 h. (**F**) Representative images of GFAP (green) and BrdU (red) immunostaining in the PBS and M2-sEV groups 14 days after tMCAO. Scale bar = 50 µm. (**G**) The number of GFAP^+^/BrdU^+^ cells in the PBS, M2-sEV, and miR-124-kd groups 14 days after tMCAO (n = 15). Data are presented as mean ± SEM. **p < 0.01, *p < 0.05.

To further verify whether M2-sEV miR-124 attenuated glial scar formation after tMCAO, we injected M2-sEVs with miR-124-kd treatment into the tMCAO mouse brain to examine glial scar formation using GFAP immunostaining. The results showed that M2-sEV miR-124-kd blocked the inhibitory effect of M2-sEVs on glial scar formation after tMCAO and that scar area and maximal thickness increased in the miR-124-kd group compared to that in the M2-sEV group** (Figure 6A-C)**.

To examine whether M2-sEV miR-124 regulated the migration and proliferation of astrocytes, we performed the scratch assay and BrdU immunostaining again. The results of the scratch assay showed that the scratch area increased in the OGD+miR-124-kd group compared to that in the OGD+M2-sEV group** (Figure 6D-E)**. BrdU immunostaining showed that the number of BrdU^+^/GFAP^+^ proliferating astrocytes increased in the miR-124-kd group compared to that in the M2-sEV group** (Figure 6D-E),** suggesting that miR-124 played a critical role in regulating astrocyte migration and proliferation after tMCAO.

### M2-sEVs blocked the STAT3 signal pathway through miR-124 in the ischemic mouse brain

**Figure 7:**
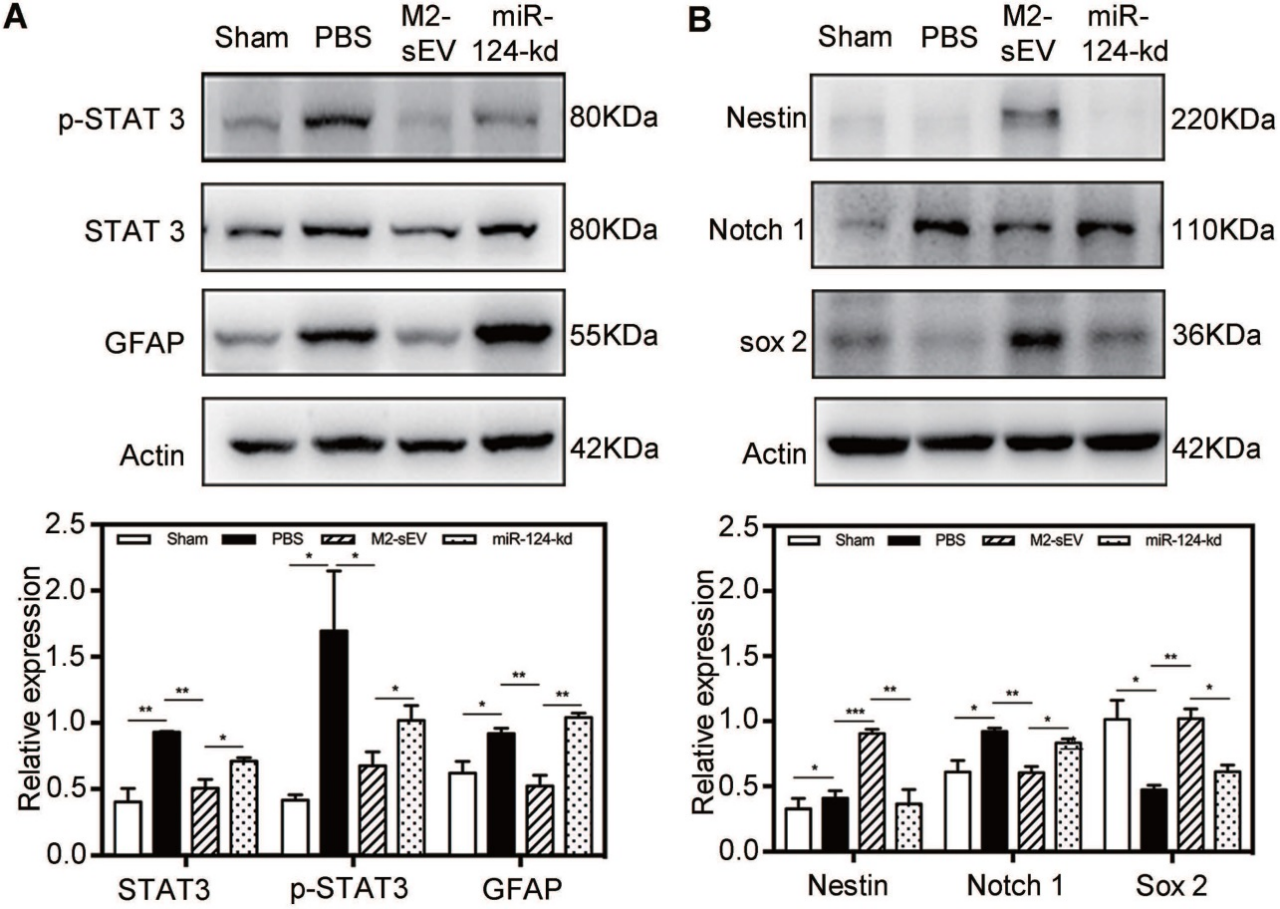
** M2-sEV blocked the STAT3 signal pathway through miR-124 in the mouse brain after tMCAO.** (**A**) Western blotting analysis and quantification of p-STAT3, STAT3, and GFAP expression in the sham, PBS -, M2-sEV -, and miR-124-kd groups after tMCAO (n = 15). (**B**) Western blotting analysis and quantification of the expression of nestin, Notch 1, and Sox 2 in the sham, PBS, M2-sEV, and miR-124-kd groups 7 days after tMCAO (n = 15). Data are presented as mean ± SEM. *p < 0.05, **p < 0.01.

To investigate the mechanism of miR-124-mediated inhibition of glial scar formation after tMCAO, we scanned the miRbase and found that STAT3 is one of the miR-124 target proteins. Accordingly, we performed western blot analysis to examine the expression of STAT3, p-STAT3 (the activated form of STAT3), and GFAP in the peri-infarct area at 7 days after ischemia. The results showed that STAT3 and p-STAT3 expression significantly increased in the brain of tMCAO mice. After M2-sEV treatment, the expression of STAT3 and p-STAT3 decreased, but was partially upregulated after miR-124-kd M2-sEV treatment **(Figure 7A)**. The downstream protein of p-STAT3 is GFAP, whose expression increased in active astrocytes after ischemia. Its expression was downregulated after M2-sEV treatment and partially upregulated in the miR-124-kd M2-sEV treated group. These results suggested that M2-sEV blocked the STAT3 pathway of astrocytes *via* miR-124.

Furthermore, we also analyzed the expression of the following transformation-related proteins using western blotting: nestin, Sox2**,** and Notch 1** (Figure 7B)**. Notch 1 activation is known to promote the differentiation of progenitor cells into astrocytes 31. Our results showed that the expression of Notch 1 increased in the brain of tMCAO mice, while its expression significantly decreased in the M2-sEV group after tMCAO. miR-124-kd M2-sEV treatment partially increased Notch 1 expression in the ischemic mouse brain. Both Sox2 and nestin expression were increased in the M2-sEV group after tMCAO compared to that in the control group, and their expression was partially reversed after miR-124-kd M2-sEV treatment **(Figure 7B)**. These results suggested that M2-sEVs promoted a phenotype transition from astrocytes to neural progenitors through miR-124.

### M2-sEVs reduced mouse atrophy volume and improved neurobehavioral outcomes through miR-124 after tMCAO

**Figure 8:**
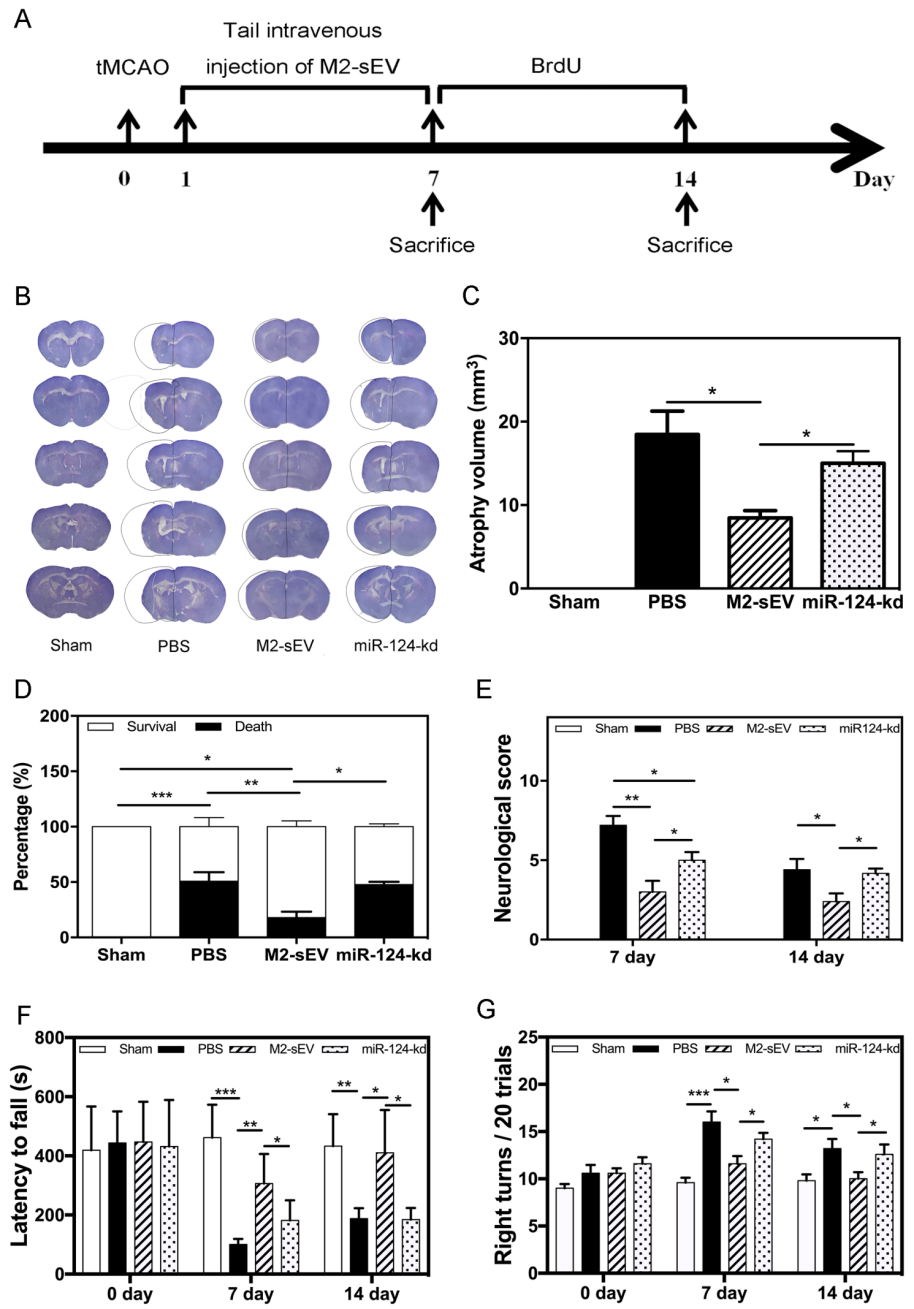
** M2-sEVs reduced atrophy volume and improved neurobehavioral outcomes through miR-124 after ischemia in mice.** (**A**) Diagram of experimental design. Animal sacrifice time points were 7 days or 14 days after tMCAO. (**B**) Cresyl violet staining for atrophy volume of brain sections in the three groups 14 days after tMCAO. The dashed line shows the original size of the ischemia side. (**C**) Atrophy volume in the sham, PBS, M2-sEV, and miR-124-kd groups 14 days after tMCAO (n = 36). (**D**) Survival percentage in the sham, PBS, M2-sEV, and miR-124-kd groups after tMCAO. (**E**) Neurological scores in the sham, PBS, M2-sEV, and miR-124-kd groups after tMCAO. (**F**) Rotarod test results in the sham, PBS, M2-sEV, and miR-124-kd groups after tMCAO. (**G**) Right turns test results in the sham, PBS, M2-sEV, and miR-124-kd groups after tMCAO. Data are presented as mean ± SEM. *p < 0.05, **p < 0.05, ***p < 0.005.

To identify the beneficial role of M2-sEV miR-124 on neurological function after tMCAO, we designed animal behavior experiments and examined the atrophy volume, survival rate, behavior, and neurobehavioral outcomes in the sham, PBS, M2-sEV, and miR-124-kd groups **(Figure 8A)**. The results showed that M2-sEVs reduced brain atrophy volumes at 14 days after tMCAO. miR-124-kd M2-sEV treatment attenuated the effects of M2-sEVs on the tMCAO mouse brain **(Figure 8B-C)**. We also found that M2-sEVs improved the survival rate of ischemic mice, which dropped immediately after miR-124-kd M2-sEV treatment **(Figure 8D)**. Next, we examined the neurological outcomes for all mice at 7 and 14 days after tMCAO using the mNSS system. The results showed that the neurological score in the M2-sEV group was lower compared to those in the control and miR-124-kd groups **(Figure 8E)**. To further examine the behavioral outcomes among all groups, we performed the rotarod test and corner tests at 7 and 14 days after tMCAO. We found that muscular coordination ability increased in the M2-sEV group compared to that in the control and miR-124-kd groups **(Figure 8F-G)**. These results suggested that M2-sEVs attenuated mouse behavioral deficits after tMCAO and that miR-124-kd M2-sEV treatment blocked the effects of M2-sEVs on behavioral deficits in tMCAO mice. Taken together, we found that M2-sEVs could reduce mouse brain atrophy volume and improve neurobehavioral outcomes through miR-124 after tMCAO.

## Discussion

In the present study, we used BV2 cells instead of primary microglia to harvest sEVs, which was due to sEV production. Our study demonstrated for the first time that sEVs derived from M2 BV2 cells reduced glial scar formation and improved functional recovery after tMCAO in mice, suggesting that microglia could affect astrocyte function *via* sEVs during brain injury. Specifically, we showed that first, the width of glial scars decreased after M2-sEV treatment. Second, M2-sEVs decreased the number of astrocyte processes and the length of the longest process in the ischemic mouse brain. Third, M2-sEVs attenuated astrocyte proliferation and migration both *in vitro* and *in vivo*. Fourth, reduced glial scar formation correlated with improvements in neurological function.

sEVs have been regarded as an efficient way of intercellular signal transfer due to their intrinsic features, including structural stability, inherent targeting properties, and the ability to overcome natural barriers 32, 33. In the CNS, all cell types have been shown to release extracellular vesicles 34 that can either be taken up by neighboring cells or released into the cerebrospinal fluid and blood 35-37. Glia and neurons can make long-distance contact by releasing and receiving sEVs 35, 38-40. In particular, microglia have been shown to largely rely on mobile vesicles or sEVs to communicate with other cells across distant brain regions 41. Furthermore, the contents of sEVs derived from microglia might differ, depending on different stimuli, and have detrimental or beneficial effects 42, 43. Microglia-derived sEVs can mediate neuroinflammation by secreting pro-inflammatory mediators such as tumor necrosis factor-α, IL-1β, and miR-155 in traumatic brain injury 44, 45; they are also known to spread the disease by tau propagation in Alzheimer's disease 46. Microglial exosomal miR-124-3p inhibited neuronal inflammation and promoted neurite outgrowth in scratch-injured neurons, which could contribute to the neurological outcome and inhibit neuroinflammation in mice with traumatic brain injury 47. miR-124 enriched exosomes increased the number of M2 microglia and improved neurogenesis and functional recovery by inhibiting the toll-like receptor 4 pathway after brain injury 48. Our study found that sEVs mediated signal transduction from M2 microglia to astrocytes and that M2-sEVs inhibited glial scar formation. Further studies are required to obtain direct evidence for sEV transfer from microglia to astrocytes *in vivo*.

We also found that M2-sEVs were enriched in miR-124, which is the most abundant miRNA in the brain. In addition, miR-124 derived from neuronal sEVs could mediate intercellular communication between neurons and microglia 49. STAT3, an miRNA-124 target 20, plays a critical role in GFAP expression 50. It is a well-known transcription factor that can regulate astrogliosis and scar formation after spinal cord injury 51. Our present M2-sEV miRNA microarray results showed that miRNA-124 was the only miRNA that was highly expressed in the CNS and could directly target STAT3 19. In addition, our results demonstrated that miR-124 derived from M2-sEVs inhibited glial scar formation and astrocyte proliferation and migration by reducing the expression of STAT3 and p-STAT3. miR-124 is also known to promote proliferation and differentiation of progenitor cells to regulate regeneration of the functional brain and the visual system by modulating the Notch pathway 52, 53. Notch 1 is a canonical factor in CNS development, and attenuated Notch1 signaling was found to be necessary for neurogenesis transition from striatal activated astrocytes after stroke 54, and blocking Notch1 or increasing Sox2 levels could trigger a latent neurogenic program in astrocytes 55, 56. Striatal astrocytes transdifferentiate into functional mature neurons, which participate in the remodeling of functional neural networks after tMCAO 57. Our results showed that M2-sEV treatment decreased Notch 1 and increased Sox2 expression in activated astrocytes, suggesting that M2-sEVs might potentially be useful for astrocyte reprogramming to neuronal progenitor cells after ischemic stroke. miR-124 might mediate this process, but how miR-124 affects Notch1 and Sox2 expression needs to be investigated in the future. In this study, we also found nestin and Sox2 expression mainly in the peri-infarct areas of the brain following tMCAO. Nestin expression did not change 14 days after tMCAO, which is consistent with a previous study showing that nestin expression was markedly upregulated at 7 days after spinal cord injury, but later declined 58. These results suggest that nestin expression in progenitors may last for a relatively short time after injury. Furthermore, cells co-expressing GFAP and nestin may further differentiate into progenitor or mature cells after 7 days of tMCAO, which needs to be investigated in the future.

In conclusion, our study demonstrated that M2-sEVs reduced glial scar formation *via* miR-124, which inhibited astrocyte proliferation and migration by reducing the expression of STAT3 as well as p-STAT3. At the same time, sEV miR-124 might be involved in astrocyte reprogramming to progenitor cells by decreasing Notch1 and increasing Sox2 expression.

## Figures and Tables

**Table:** The sequences of the mRNA oligonucleotide primers

Gene	Forward and Reverse Sequences
Mouse IL-1	F: CGCAGCACAGCACATCAACAAGAGC
	R: TGTCCTCATCCTGGAAGGTCCACG
Mouse Arginase	F: CTCCAAGCCAAAGTCTTAGAG
	R: AGGAGCTGTCATTAGGGACATC
Mouse GAPDH	F: AGGTCGGTGTGAACGGATTTG
	R: TGTAGACCATGTAGTTGAGGTCA

## Abbreviations

GFAP: glial fibrillary acidic protein
IL: interleukin
sEVs: small extracellular vesicles
M2-sEV: Type 2 microglia derived small extracellular vesicles
BrdU: bromodeoxyuridine
STAT3: signal transducer and activator of transcription 3
p-STAT3: phosphorylated STAT3
miR: microRNA
miR-124-kd: miR-124 knockdown
tMCAO: temporary middle cerebral artery occlusion
PBS: phosphate-buffered saline
DMEM: Dulbecco's Modified Eagle medium
FBS: fetal bovine serum
PFA: paraformaldehyde
mNSS: modified neurological severity score
BSA: bovine serum albumin
DAPI: 4, 6-diamidino-2-phenylindole
OGD: oxygen glucose deprivation
TEM: transmission electron microscopy
NTA: nanoparticle tracking analysis
IF: intermediate filament
CNS: central nervous system
SEM: standard error of mean
